# Ketosis and migraine: a systematic review of the literature and meta-analysis

**DOI:** 10.3389/fnut.2023.1204700

**Published:** 2023-06-12

**Authors:** Lenycia de Cassya Lopes Neri, Cinzia Ferraris, Guido Catalano, Monica Guglielmetti, Ludovica Pasca, Elena Pezzotti, Adriana Carpani, Anna Tagliabue

**Affiliations:** ^1^Ketogenic Metabolic Therapy Laboratory, Department of Public Health, Experimental and Forensic Medicine, University of Pavia, Pavia, Italy; ^2^Faculty of Medicine, Department of Pediatrics, University of São Paulo, São Paulo, Brazil; ^3^Laboratory of Food Education and Sport Nutrition, Department of Public Health, Experimental and Forensic Medicine, University of Pavia, Pavia, Italy; ^4^Department of Child Neurology and Psychiatry, Istituto di Ricerca e Cura a Carattere Scientifico Mondino Foundation, Pavia, Italy; ^5^Department of Brain and Behavioral Sciences, University of Pavia, Pavia, Italy

**Keywords:** migraine disorders, ketogenic diet, ketosis, systematic review, meta-analysis

## Abstract

**Introduction:**

Headaches are a prevalent disorder worldwide, and there is compelling evidence that certain dietary interventions could provide relief from attacks. One promising approach is ketogenic therapy, which replaces the brain's glucose fuel source with ketone bodies, potentially reducing the frequency or severity of headaches.

**Aim:**

This study aims to conduct a systematic review of the scientific literature on the impact of ketosis on migraine, using the Preferred Reporting Items for Systematic Reviews and Meta-Analyses (PRISMA) method.

**Results:**

After a careful selection process and bias evaluation, 10 articles were included in the review, primarily from Italy. The bias assessment indicated that 50% of the selected articles had a low risk of bias in all domains, with the randomization process being the most problematic domain. Unfortunately, the evaluation of ketosis was inconsistent between articles, with some assessing ketonuria, some assessing ketonemia, and some not assessing ketosis levels at all. Therefore, no association could be made between the level of ketosis and the prevention or reduction of migraine attacks. The ketogenic therapies tested in migraine treatments included the very low-calorie ketogenic diet (VLCKD, *n* = 4), modified Atkins diet (MAD, *n* = 3), classic ketogenic diet (cKDT, *n* = 2), and the administration of an exogenous source of beta-hydroxybutyrate (BHB). The meta-analysis, despite reporting high heterogeneity, found that all interventions had an overall significant effect (*Z* = 9.07, *p* < 0.00001; subgroup differences, Chi2 = 9.19, dif = 3, *p* = 0.03; *I*^2^, 67.4%), regardless of the type of endogenous or exogenous induction of ketosis.

**Conclusion:**

The initial findings of this study suggest that metabolic ketogenic therapy may provide some benefit in treating migraines and encourage further studies, especially randomized clinical trials with appropriate and standardized methodologies. The review strongly recommends the use of the adequate measurement of ketone levels during ketogenic therapy to monitor adherence to the treatment and improve knowledge of the relationship between ketone bodies and efficacy.

**Systematic review registration:**

https://www.crd.york.ac.uk/prospero/, identifier: CRD42022330626.

## 1. Introduction

Headache is one of the most common disorders in the general population, and it is frequently diagnosed in childhood (1), with a prevalence of 54.4–58.4% in children and adolescents (2). It is the third biggest cause of disability across the world (3), and in 2019 it was ranked 14th overall for global causes of disability-adjusted life years, rising to 10th place for females and ranked 2nd and 5th among individuals aged 10–24 and 25–49, respectively (4). According to the International Classification of Headache Disorders 3rd edition (ICHD-3) (5), headache is classified into primary headache, with no underlying organic causes, and secondary headache (6). Primary headache onset often occurs in childhood or adolescence and its prevalence grows with age, heavily impacting a child's quality of life, in particular on school performance, sports, and social activities (3). Tension-type headache (prevalence of 20–25%) is the most common cause of primary headache, followed by migraine (7). Migraine prevalence is ~15% in the general population, with a peak between 35 and 39 years (8). Moreover, migraine affects 7.7–9.1% of children, and girls are the most impacted (2). According to the ICHD-3, the three main categories of migraine are migraine without aura, migraine with aura, and chronic migraine. Aura consists of reversible focal neurologic symptoms (visual scintillations, scotoma, and, less often, spreading hemisensory symptoms or speech dysfunction) that develop gradually over a period of 5–60 min (or less) followed by a subsequent headache, typically unilateral, pulsatile, and aggravated by physical activity. Common accompanying symptoms are nausea, vomiting, photophobia, and phonophobia (5).

Migraine is a neurovascular disease that tends to run in families and likely has a genetic basis (9). The most widely accepted etiopathogenetic theory is Moskowitz's trigeminovascular hypothesis (10, 11). According to this theory, patients are predisposed to migraine attacks due to an over-sensitization of the trigeminal and trigemino-cervical neurons, which is associated with a lowered threshold of activation of nociceptive terminals by vasoactive peptides, the most important of which is CGRP (12). In addition, there is strong evidence to suggest that migraine is a brain energy deficit syndrome: several magnetic resonance spectroscopy studies have highlighted that the brains of migraine sufferers experience an energy deficit during attacks, likely in response to hypometabolism and increased oxidative stress in the brain (13). Current prophylactic therapies for migraine often suffer from a lack of specificity, poor tolerability, potential side effects, and limited efficacy, leading to unsatisfactory results in a large proportion of patients (14). Although several promising monoclonal antibodies have been developed for the adult population and are now being implemented in the pediatric population, most of them are not currently available in clinical practice. In parallel with the development of novel therapeutics for migraine, there is a growing body of literature on the use of neuromodulatory non-pharmacologic approaches, such as nutraceuticals (e.g., riboflavin, coenzyme Q10, magnesium, vitamin D, and melatonin) or behavioral therapies (15).

Nutrition is a widely discussed environmental factor that may affect the course of migraine (16). While there is a debate over how certain foods can act as favorable or protective factors in relation to migraine attacks and the pro-inflammatory state, it is accepted that migraine is sensitive to diet, including food amount and meal timing, and that some dietary ingredients can trigger migraine attacks (16). A long list of foods involved in the mechanism of migraine has been identified, such as chocolate, citrus fruits, nuts, ice cream, tomatoes, onions, dairy products, alcoholic beverages, coffee, caffeine, monosodium glutamate, histamine, tyramine, phenylethylamine, nitrites, aspartame, sucralose, and gluten. Some foods or ingredients can trigger headaches only in subgroups of patients (e.g., celiac groups), while others can cause migraines in case of abstinence (such as caffeine) (17). Even when these elements are correctly identified, the use of diets with certain food restrictions remains controversial (17). Low-fat or weight-loss dietary interventions for migraine have so far been inconclusive. The mechanisms through which nutrition could impact migraine could be related to decreasing inflammation, such as with high omega-3/low omega-6 diets, which can bring beneficial effects. Nonetheless, more studies are needed (18).

In recent years, the use of high-fat low-carbohydrate diets has gained popularity as a possible treatment for migraine. The International Ketogenic Diet Study Group cites migraine as one of the neurological diseases that can potentially benefit from ketogenic dietary treatment (KDT) (19). Interestingly, the first report of using KDT for migraine appeared in 1928 (20), only a few years after the diet's first use for epilepsy. This study obtained poor results (some relief in nine of the 23 adult patients), but the author reported being encouraged enough to continue with the high-fat method. In the last few years, several studies described in a review by Caminha et al. (21) have investigated the potentially protective effects of ketosis-inducing diets in migraine. Ketogenic diet therapies may affect migraine in several ways: (i) by replacing brain fuel from glucose to ketone bodies (KBs); (ii) through the positive influence of systemic ketosis on pathways of migraine pathophysiology; (iii) as signaling molecules, KBs could increase mitochondrial functioning, reduce oxidative stress, alter cerebral excitability, change cortical spreading depression, reduce systemic inflammation, and change the gut microbiome (22). The concept of a gut-brain axis can stimulate the use of probiotics for several neurological diseases, but studies investigating probiotics for migraine are limited and often mix the application of probiotics with other components (17).

Based on the protective effects of ketone bodies, an interesting question that has yet to be answered is whether the levels of ketosis, measured by circulating levels of beta-hydroxybutyrate (BHB), are related to the efficacy of migraine attack prevention. To achieve high systemic ketosis, it is necessary to follow a diet that is high in fat, moderate in protein, and very low in carbohydrates, such as the classical ketogenic diet with 3:1 or 4:1 ketogenic ratios. However, there are physiological differences between children and adults, and even when treated with diets at the same ketogenic ratio, adults have much lower blood ketone levels than children (22, 23). Furthermore, the classic ketogenic diet is more burdensome for patients, requiring a strict dietary plan and weighing of foods to the gram. Alternative dietary approaches, such as the medium-chain triglycerides diet, modified Atkins diet, and low glycemic index therapy, have been developed to allow for greater flexibility for patients in epilepsy treatment. From a clinical perspective, it is of paramount importance to establish target levels of ketosis that are sufficient to maintain the beneficial effect on migraine while limiting dietary restrictions.

This systematic review and meta-analysis are an initial effort to systematize information on the efficacy and tolerability of different ketogenic diets and levels of ketosis in the prevention of migraine in children, adolescents, and adults.

## 2. Materials and methods

This systematic review and meta-analysis was conducted following the Preferred Reporting Items for Systematic Reviews and Meta-Analyses (PRISMA) guidelines (24). A comprehensive search was performed in electronic databases, including PubMed/Medline, Scopus, Web of Science, LILACS, Science Direct, and the Cochrane Library. The search was limited to articles published in the last 10 years, and only studies published in English, Italian, Portuguese, or Spanish were included.

To investigate the effect of ketosis on migraine, both clinical trials and observational studies were included regardless of whether they were controlled or randomized. The study protocol was registered on the PROSPERO platform (registration number: CRD42022330626).

### 2.1. Literature search

An electronic search was conducted using subject index terms such as “migraine,” “migraine disorders,” “headache,” “headache disorders,” “cephalalgia,” and “cephalalgia” in combination with keywords such as “ketones,” “3-hydroxybutyric acid,” “ketone bodies,” “acetoacetates,” “ketosis,” “ketoacidos^*^,” “metabolic keto^*^,” “acetonemi^*^,” “ketonemi^*^,” “ketoacidemi^*^,” “ketonuri^*^,” “ketoaciduri^*^,“ or “acetonuri^*^”. The final search strategy is described in detail in the Supplementary material. In addition, gray literature was searched using Google Scholar. Some newer references from more up-to-date studies or references found in previous review articles were included manually. The populations of interest were adults, adolescents, and children, and the comparison group was any control diet or placebo group. Detailed inclusion and exclusion criteria are described in Table 1.

**Table 1:** PICOS inclusion and exclusion criteria.

**PICOS criteria**	**Inclusion criteria**	**Exclusion criteria**
Population	Children, adolescents, and adults with a diagnosis of migraine	Individuals without migraine
Intervention	Achievement of ketosis by: —production of endogenous ketone bodies induced by ketogenic dietary therapies (CKD, MCT, LGI, MAD, or VLCKD) OR —administration of exogenous ketone bodies (EK)	Ketosis unrelated to ketogenic diets or exogenous ketone body administration (e.g., ketosis in diabetes)
Comparison	Comparison patients who remained on their usual diet, on placebo treatment, or on pharmacologic treatment; other interventions; without a comparator	Not applicable
Outcomes	Reduction in the frequency and intensity of migraine episodes	Unrelated to migraine episodes
Types of studies included	Randomized controlled trials; uncontrolled observational studies	Full text not available; without the outcomes of interest; not human studies; reviews, opinion articles, guidelines, letters, editorials, comments, case reports and case series, news, conference abstracts, theses, and dissertations; and *in vitro* or animal studies
Research question	What is the efficacy of the ketosis for the prevention or attenuation of migraine?	

### 2.2. Study selection

The research and study selection were carried out independently by two authors (LCLN and GC) using Rayyan software (25) in two steps: (1) reading the titles and abstracts, and (2) evaluating the complete articles selected in the previous stage and including other studies present in the references of the selected articles. The decision to include the articles was based on the PICOS strategy [Population (P), children and adults; Intervention (I), ketosis/ketone bodies; Control (C), placebo; Outcome (O), relief in migraine symptoms; Study type (S), clinical trials]. In cases of disagreement, a third author (MG) reviewed the full-text articles to decide. The articles found in the electronic databases were organized using the Mendeley reference manager. Google Scholar was used to search for gray literature.

### 2.3. Study quality

The risk of bias was assessed using the RoB 2.0 Cochrane tool (26), which checked five domains: randomization process, deviations from intended interventions, missing outcome data, measurement of the outcome, and selection of the reported result. After selecting articles, the quality of evidence was checked using the Mixed Methods Appraisal Tool system (MMAT system) (27). Data tables were constructed based on article details, and data for generating the meta-analysis were exported to Review Manager (RevMan) 5.4 software (28).

## 3. Results

**Figure 1:**
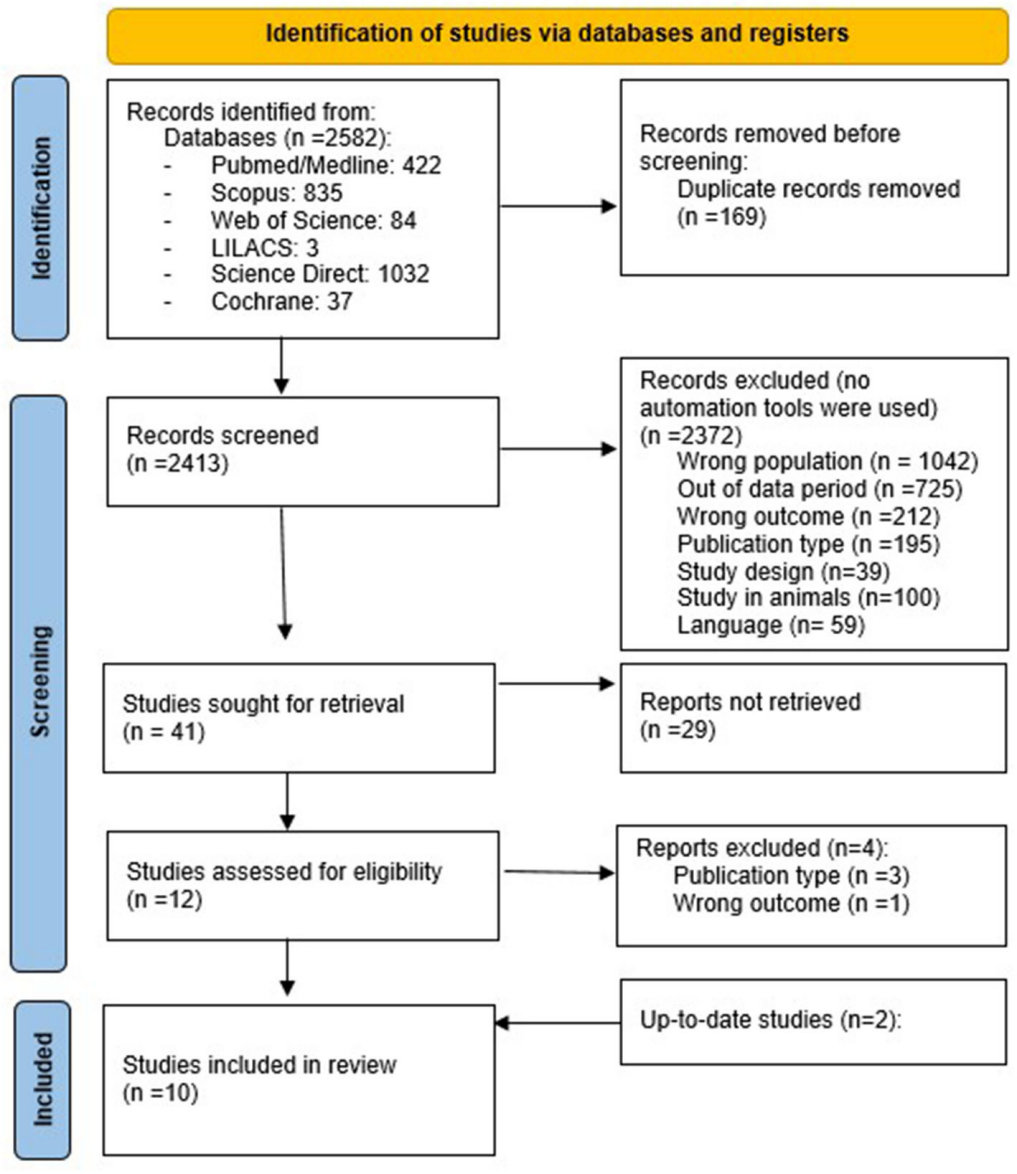
Flow chart of the selection of articles according to the PRISMA method.

A total of 2,582 articles were identified from all databases. After a systematic selection of articles that excluded duplicates (carried out by two authors), only 10 articles remained (eight conducted in Italy, one in Switzerland, and one in Australia). The article selection process is illustrated in the flow chart (Figure 1), following the PRISMA method (24).

The study population, type and duration of ketogenic therapy intervention, levels of urine or blood ketosis (if reported), outcomes, and dropout rates in the selected articles are synthesized in Table 2. All studies were conducted on adults affected by migraine, except for one that concerned the treatment of cluster headaches. Half of the selected articles used an interventional open-label trial design, and only one article used retrospective observational data. The different ketogenic dietary treatments used in the selected studies are described in the following section.

**Table 2:** Description of all selected articles.

**References**	**Type of study**	**Population (*n*, age, sex, nutritional status, diagnosis)**	**Intervention (KDT type, duration)**	**Ketosis assessment**	**Outcomes**	**Dropout**	Quality (MMAT^*^)
Di Lorenzo et al. (29), Italy	Prospective observational study	*n* = 96, 18–65 years old, all F, OW, migraine with or without aura. Controls: OW F without headache. Baseline migraine attacks per month (*n* ± SD): 2.91 ± 1.73.	6 months on a dietetic plan. —Participants (*n* = 45) on 4 weeks VLCKD based on food replacements, 4 weeks transitional diet with nutraceutical integrators, 4 weeks transitional diet without nutraceutical integrators, and the residual period standard diet. - Controls (*n* = 51): 6-month low-calorie standard diet.	Twice a day urine ketone dipstick tests: 4–10 mmol/L^**^ during the VLCKD.	VLCKD: frequency and tablet use reduced after the first month of the diet (*p* < 0.0001). Transition period worsening, despite being improved compared with the baseline. Control: number of days with headaches and tablet intake reduced from month 3 (*p* < 0.0001), attack frequency reduced at month 6 (*p* < 0.0001).	*N* = 15 (16%) abandoned the study (9 at the third and 6 at the sixth month).	^***^
Di Lorenzo et al. (30), Italy	Open-label controlled interventional study	*n* = 18, 19–54 years old, 16F/2M, NW and OW, migraine with or without aura. Controls: healthy volunteers with comparable BMI. Baseline migraine attacks per month (*n* ± SD): 4.40 ± 2.70.	BMI ≥ 25: 4-week VLCKD. BMI < 25: 4-week MAD supplemented with lipid powder (MCT, LCT, and omega-3) with nutraceutical integrators.	Daily urine ketone dipstick test confirmed the presence of ketosis (not mentioned in the numeric results).	Reduction in attack frequency and duration (*p* < 0.001). No change to the first SSEP and VEP block of responses, but normalization of the interictally reduced VEP and SSEP (*p* < 0.01) habituation during the subsequent blocks.	*n* = 07 (28%) did not fulfill primary inclusion criteria.	^**^
Di Lorenzo et al. (33), Italy	Prospective open-label single-arm clinical trial	*n* = 18, 25–55 years old, 7F/11M, NW, cluster headache. Baseline migraine attacks per month (*n* ± SD): 108.17 ± 81.71.	12-week MAD supplemented with lipid powder (MCT, LCT, and omega-3) with nutraceutical integrators.	Not mentioned.	Full resolution of headache in 11/18 patients, at least 50% reduction in 4/18 patients.	*n* = 00.	^***^
Di Lorenzo et al. (34), Italy	Prospective open-label single-arm clinical trial	*n* = 18, >18 years old, 16F/2M, BMI 26.7 ± 4.6 kg/m^2^, episodic migraine without aura. Controls: HVs with a comparable BMI. Baseline migraine attacks per month (*n* ± SD): 4.70 ± 2.50.	4-week MAD supplemented with lipid powder (MCT, LCT, and omega-3) with nutraceutical integrators (ratio 1.7–2: 1).	Urine ketone dipstick on the day of testing >0.5.	Patients after KDT exhibited a reduction in attack frequency (*p* < 0.001), duration (*p* = 0.001), and disability (*p* < 0.001); normalization of the intercritical pain-related evoked potential habituation; no change in nBR; no change in BMI; HVs exhibited a physiologic habituation in the N-P amplitude slope of PREP, and both in homolateral and contralateral nBR.	*n* = 4 (18%) later determined that did not fulfill the primary inclusion criteria.	^**^
Di Lorenzo et al. (32), Italy	Single-center randomized double-blind controlled crossover phase 2 trial	*n* = 35, 18–65 years old, 29F/7M OW or obese, migraine with or without aura, prediabetes. Baseline migraine attacks per month (*n* ± SD): 4.83 ± 2.01.	4-week periods on a VLCKD or VLCnKD in a randomized order.	Urine ketone dipstick. 75.9% of patients on VLCKD positive (range 0.5–4.5 mmol/L).	4-week period VLCKD, despite inducing similar weight loss and glycemic profile, was significantly more effective than VLCnKD in preventing migraine attacks, as evidenced by a decrease in the frequency of migraine days and attacks and a >50% response rate.	*n* = 6 (17%) due to the excessive difficulty of the VLCnKD.	^*****^
Valente et al. (36), Italy	Retrospective observational study	*n* = 23, 47.22 ± 15.21 years old, 22F/1M, 10 NW, 8 OW, 5 obese, migraine. Baseline migraine attacks per month (*n* ± SD): 12.50 ± 9.50.	3 months on KDT tailored to the patient's characteristics: *n* = 4 VLCKD, *n* = 5 cKDT 2:1, *n* = 5 cKDT 1.5:1, *n* = 8 cKDT 1:1, *n* = 1 cKDT 0.5:1.	Some patients had blood measurement, but not reported because it was not systematically collected.	Reduction in monthly headache days (12.5 ± 9.5 vs. 6.7 ± 8.6; *p* < 0.001). Reduction in days of acute medication intake (11.06 ± 9.37 vs. 4.93 ± 7.99; *p* = 0.008).	*n* = 10 (30%) due to poor compliance, reported inefficacy, excessive weight loss, lost at follow-up without known reasons.	^***^
Bongiovanni et al. (31), Italy	Open-label single-arm clinical trial	*n* = 23, 18–57 years old, 21F/2M. Median BMI 26.5 (19.7–42.2), refractory chronic migraine. Baseline migraine attacks per month (days/range): 30/12–30.	3 months: BMI >30 kg/m^2^: VLCKD (800 kcal) BMI ≥ 25 kg/m^2^: LC (1,100–1,300 kcal) BMI < 25 kg/m^2^: LC (1,500–1,700 kcal) 1 month on transition phase: CHO increase: 30 g every week up to 150 g. 2 months on maintenance phase: LGIT.	Not measured.	Reduction in duration: hours per day (*p* < 0.0016) and days per month (*p* < 0.0001). Reduction in intensity of pain of each migraine episode (*p* < 0.0024). Reduction of doses of analgesics taken in a month. Decrease in weight and BMI (*p* < 0.0001).	*n* = 15 (39%) without mention of reason.	^***^
Haslam et al. (35), Australia	Pilot randomized controlled crossover trial	*n* = 16, >18 years old, 14F/2M mean BW 77.5 ± 15.9 kg mean body fat 35.6 ± 7.7% Migraine Baseline migraine attacks per month: not mentioned.	12-weeks divided into two 4-week dietary intervention periods interspersed with one washout period. cKDT: 3:1 ratio; lower ratios in order to aid compliance and retention; anti-headache diet: excluding dietary-related migraine triggers.	Urinary ketosis was measured for 81% of participants on 18 out of 28 days; average ketone level 7.2 mmol/L across the 28 days (range 2.0–14.0 mmol/L).	There were no statistically significant differences in migraine frequency, severity, or duration.	*n* = 10 (38%) due to being lost at follow-up or discontinued intervention (diet too difficult) or commenced another diet.	^***^
Putananickal et al. (37), Switzerland	Single-center randomized placebo-controlled double-blind crossover trial	*n* = 32, 18–65 years old, 37F/3M, BMI mean 23.96 ± 4.33 kg/m^2^. Episodic migraine. Baseline migraine attacks per month (*n* ± SD): 7.50 ± 2.60.	12-week treatment periods on exogenous DL-Ca-Mg-BHB supplement divided into 3 daily servings; placebo: mannitol. Ketone supplement and placebo were administered with glucose-free syrup to overshadow taste difference. No dietary intervention.	Blood ketone levels were assessed using a portable point-of-care blood ketone meter. —BHB after 40 min intake: 0.40 mmol/L (0.30, 0.50 mmol/L).	No beneficial effect in migraine frequency or intensity during BHB treatment.	*n* = 3 (22%) withdrawal of consent or due to intolerable side effects.	^*****^
Valente et al. (36), Italy	Retrospective observational study	*n* = 23, 47.22 ± 15.21 years old, 22F/1M, 10 NW, 8 OW, 5 obese, migraine. Baseline migraine attacks per month (*n* ± SD): 12.50 ± 9.50.	3 months on KDT tailored to the patient's characteristics: *n* = 4 VLCKD, *n* = 5 cKDT 2:1, *n* = 5 cKDT 1.5:1, *n* = 8 cKDT 1:1, *n* = 1 cKDT 0.5:1.	Some patients had blood measurement, but not reported because it was not systematically collected.	Reduction in monthly headache days (12.5 ± 9.5 vs. 6.7 ± 8.6; *p* < 0.001). Reduction in days of acute medication intake (11.06 ± 9.37 vs. 4.93 ± 7.99; *p* = 0.008).	*n* = 10 (30%) due to poor compliance, reported inefficacy, excessive weight loss, lost at follow-up without known reasons.	^***^
Lovati et al. (38), Italy (38)	Open-label single-arm clinical trial	First study: KDT group: *n* = 13, 36.9 ± 12.9 years old 11F/2M, BMI: 29.1 ± 5.4. LC group: *n* = 8, 50.9 ± 10.8 years, all F, BMI 28.9 ± 6.3. Baseline migraine attacks per month (*n* ± SD): 19.10 ± 6.50. Second study: KDT group: *n* = 26, 24F/2M. LC group: *n* = 6, 5F/1M.	3 weeks: KDT: normocaloric or hypocaloric MAD with MCT (10 g/d). LC: < 40% of CHO.	Carried out only in Study 1: Urinary ketone levels were measured in all patients, independently if KDT or LC: ketones were labeled as absent (*n* = 97), low (+; *n* = 54), moderate (++; *n* = 135), and high (+++; *n* = 70).	Based on the first study: relationship between ketone production and effect on headache; the higher the ketones, the lower the migraine frequency (chi-square *p* = 0.0073).	First study: *n* = 5 (19%) retired the consent after learning about the dietary changes. Second study: *n* = 0.	^***^

KDT, ketogenic diet therapies; BW, body weight; BMI, body mass index; OW, overweight; NW, normal weight; F, female; M, male; SD, standard deviation; CHO, carbohydrates; LC, low-carb diet; VLCKD, very low-calorie ketogenic diet; VLCnKD, very low-calorie non-ketogenic diet; MAD, modified Atkins diet; LGI, low glycemic index diet; cKDT, classical ketogenic diet therapy; BHB, beta-hydroxybutyrate; SSEPs, somatosensory-evoked potentials; VEP, visual-evoked potentials; MCT, medium-chain triglycerides; LCT, long-chain triglycerides; HVs, healthy volunteers; PREP, pain-related evoked potentials; nBR, nociceptive blink reflex; N-P, negative-positive.

^*^MMAT: Mixed Methods Appraisal Tool system (27): from ^*^ (1 star = lower quality) up to ^*****^ (5 stars = higher quality).

^**^Conversion for ketones measured in urine (acetoacetate): 10 mg/dL is equivalent to 1 mmol/L.

### 3.1. Ketogenic therapies tested in migraine treatment

#### 3.1.1. Very low-calorie ketogenic diet

Most articles (four out of 10) used a Very Low-Calorie Ketogenic Diet (VLCKD) (29–32). In these studies, the diet consisted of a semi-starvation regimen ( ≤ 800 kcal) composed of low-carbohydrate (about 30 g/day carbohydrates), low-fat (15–20 g lipids), and a normal-protein (1.0–1.5 g/kg of desired weight proteins) amount. Patients were instructed to avoid rice, grains, cereals and derivatives (bread, pasta, crackers, cookies, etc.), legumes, starchy vegetables (potatoes, corn, and green peas), fruits, and dairy products other than cheese, cream, or butter. The diet plans of the studies included Italian dietary products developed by industries. Owing to dietary restrictions, nutraceutical integrators were prescribed. Salads were allowed *ad libitum*, dressed with a spoonful of olive oil. After the VLCKD period, patients progressively reintroduced carbohydrates from breakfast to dinner to wean themselves from ketogenesis. At the end of the transition period or for the control group, patients received a standard diet (SD) or low glycemic index diet (LGID). In these cases, the SD was considered a low-calorie diet (1,200–1,500 kcal) subdivided into five daily meals with the following profile: 46% of total energy from carbohydrates, 24% as protein, and 30% as total fat (< 8% saturated fat). LGID consisted of exchanging the provision of carbohydrates into a low glycemic index (< 50) carbohydrate profile.

#### 3.1.2. Modified atkins diet

Another ketogenic diet therapy approach used in three articles (30, 33, 34) was the modified Atkins diet (MAD). In these studies, the approach consisted of a low-carbohydrate (~15 g/day), normal/low-protein (about 0.7–1.2 g/kg/day), and high-fat (approximately little more than the weight of carbohydrates and proteins together) diet prepared from common foods. Most articles allowed no more than 10 g of carbohydrates per day in the first month (after that, up to 20–30 g/day) subdivided into three regular-size meals a day or four to five smaller meals. Each meal consisted of a liberal combination of fat and protein in the form of fish, shellfish, poultry, red meat, eggs, or low-carbohydrate and high-fat cheese, dressed with butter, heavy whipping cream, mayonnaise, olive oil, and other vegetable oils. Almonds, nuts, and oilseeds were suitable as snacks. Salad vegetables were suggested twice per day, dressed with oil, mayonnaise or sour cream, vinegar (without added sugars, no balsamic), and salt. Leafy vegetables, up to 200 g per portion, were permitted, while other vegetables were limited. All spices (without added sugar) were allowed.

#### 3.1.3. Classic ketogenic diet

Only two articles (35, 36) used classical ketogenic diet therapy (cKDT), which is commonly used for refractory epilepsy. This ketogenic therapy was presented in individualized meal plans, calculated by a dietitian according to ratios of grams of total fat to carbohydrate plus protein (3:1, 2:1, and 1:1). Haslam et al. (35) provided meal plans for a rotating 7-day 1,600 (females) or 1,800 (males) kcal/day, presented as three meals per day. Participants had the option to snack on additional items from an approved list with a 3:1 ratio of total fat to combined carbohydrates plus protein (e.g., 30 g of nuts) or choose extra meals *ad libitum* from the meal plan to manage hunger and/or fatigue as needed. Participants were advised that they could flavor the meals with herbs, spices, salt, and pepper, as desired, but should avoid using pre-packaged sauces to moderate total carbohydrate intake. They were instructed to abstain from alcohol and not deviate from the meal plans. Black tea, coffee, and artificially sweetened beverages, such as diet soft/soda drinks, were recommended *ad libitum*. All participants started on a 3:1 diet plan and were monitored by a dietitian, with weekly follow-ups via email or telephone. If a participant indicated he/she was struggling to adhere to the initial 3:1 diet due to food portion sizes or was not tolerating the high-fat content or carbohydrate restriction, he/she was downgraded onto the 2:1 and then 1:1 to aid compliance and retention in the study, while achieving ketosis.

#### 3.1.4. Other approaches

One study used a low-carb (LC) approach with a low-calorie but high-protein diet (31), while another study (37) did not use a specific dietary approach and instead relied solely on an exogenous source of beta-hydroxybutyrate (BHB).

All the aforementioned articles can be found in detail in Table 2.

### 3.2. Efficacy on migraine

The studies by Di Lorenzo et al. (29, 30, 33, 34) and Bongiovanni et al. (31) demonstrated a positive effect on the frequency and intensity of migraine attacks. Some studies reported that the percentage of patients achieving a decrease in the number of migraine attacks (generally of at least 50%) ranged from 58 to 83% of patients (31, 32, 36, 38), and full resolution of attacks was reported in 63% of patients in the study by Di Lorenzo et al. (33) (data not shown). There were five single-arm intervention studies (29–31, 33, 34) and one randomized trial (32).

On the other hand, two other clinical trials (35, 37) did not reach statistically significant results on the efficacy of ketogenic therapy. Unfortunately, owing to a shortage of included studies and the lack of clinical data availability, a correlation of KD efficacy on migraine type was not feasible.

### 3.3. Ketosis assessment

The assessment of ketosis varied greatly among the articles, with ketonemia measured in only two articles (36, 37) and ketonuria measured in six articles (29, 30, 32, 34, 35, 38).

In studies using VLCKD, Bongiovanni et al. (31) did not measure ketone levels, while Di Lorenzo et al. used ketonuria as the preferred method of ketosis monitoring. The authors described that a daily urine dipstick confirmed the presence of ketosis without reporting values in one paper (30) and values in the range of 0.5–10 mmol/L were reported in the other two studies (29, 32). Ketonuria was the preferred method of ketosis monitoring in all studies using MAD (30, 33, 34), but values were not detailed.

In the study using cKDT, Valente et al. (36) reported that many patients measured blood ketones, but the authors did not systematically collect or report any data. On the contrary, in the study by Haslam et al. (35), urinary ketosis was measured by 81% of participants in 18 out of 28 days, with an average level of 7.2 mmol/L (range 2.0–14.0 mmol/L). Putananickal et al. (37) measured blood ketones and described average values of 0.4 mmol/L 40 min after the intake of ketone supplements.

Therefore, some authors did not report the results from this assessment or did not measure it, impairing the analysis of adherence to the treatment. Surprisingly, studies that reported similar values of ketonuria (29, 35) found contrasting results on outcomes with the intervention. On the other hand, Lovati et al. (38) found an inverse correlation between ketonuria and the frequency of migraine episodes in 13 patients.

### 3.4. Tolerability

In general, all studies showed good tolerability of ketosis with some common related side effects, including constipation (32, 35, 36), fatigue, nausea (35, 36), bloating (33, 35), difficulty sleeping, dizziness, irritability, flatulence (35), muscle cramps (32), abdominal pain, excessive weight loss (36), and hair loss (33). Some studies reported no side effects of the intervention (38). An average of a 21% (0–39%) dropout rate could be a reason for the small final sample sizes in all studies. Some studies justified the high dropout rate due to difficulties with diet compliance, which is often reported as incompatible with a normal social life (31).

### 3.5. Meta-analysis

A meta-analysis was performed only with studies that had available data regarding the average number of migraine attacks per month before and after intervention, along with their standard deviation.

The meta-analysis was performed on subgroups based on the type of intervention. The VLCKD intervention had four articles (29–32), but one of them (31) did not have complete data so it was excluded. Four studies were considered for the MAD intervention (30, 33, 34, 38), and one of them (33) had more expressive results. Only one study was included for the interventions with exogenous BHB (37) and cKDT (36).

**Figure 2:**
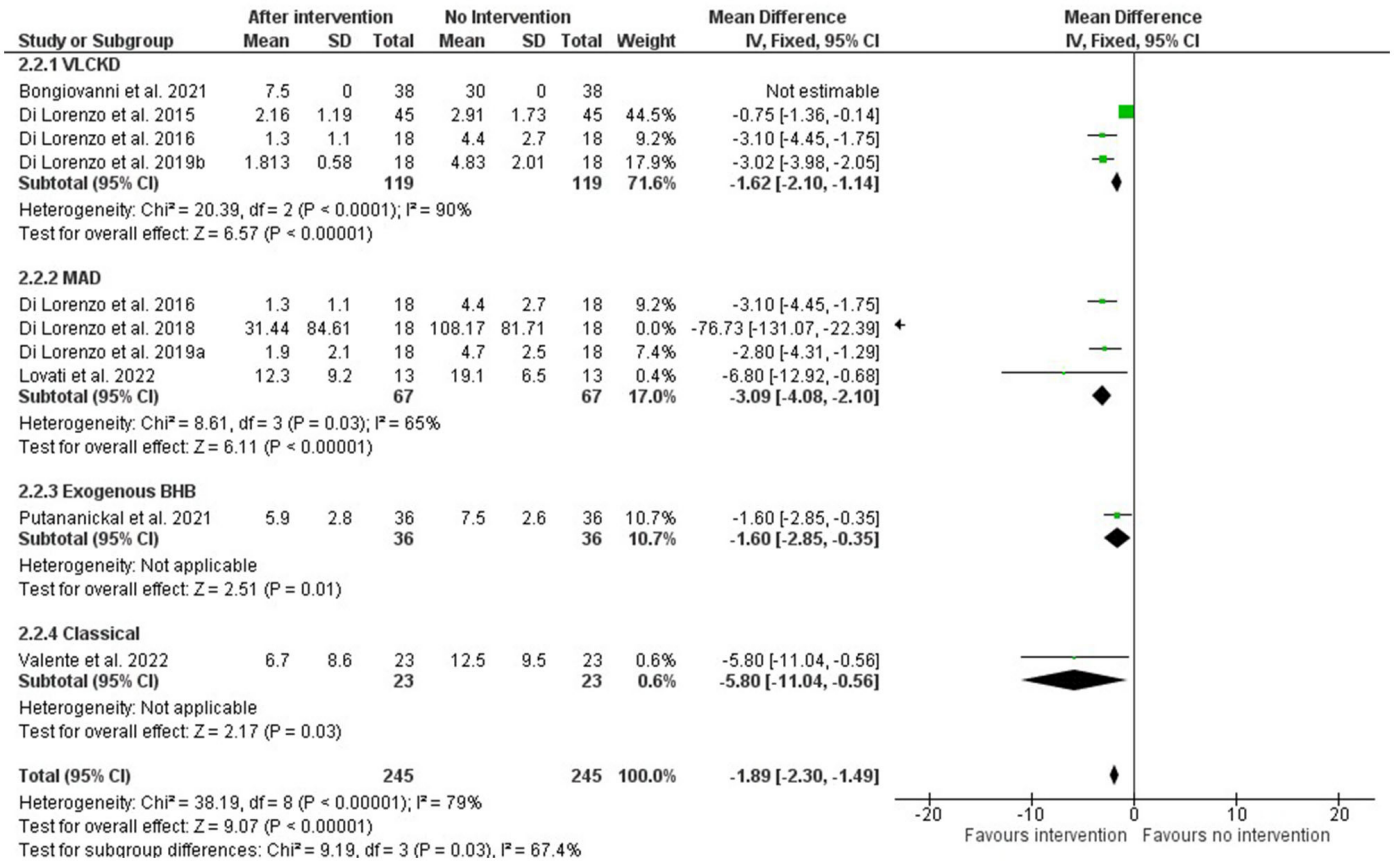
Forest plot of the meta-analysis of different types of ketosis-inducing interventions on migraine frequency attacks (number per month).

Despite high reported heterogeneity, all interventions had an overall significant positive effect, as seen in Figure 2, regardless of the type of endogenous or exogenous induction of ketosis. For the EK and cKDT approaches, as they are alone in the category, heterogeneity was not applicable.

### 3.6. Bias assessment

**Figure 3:**
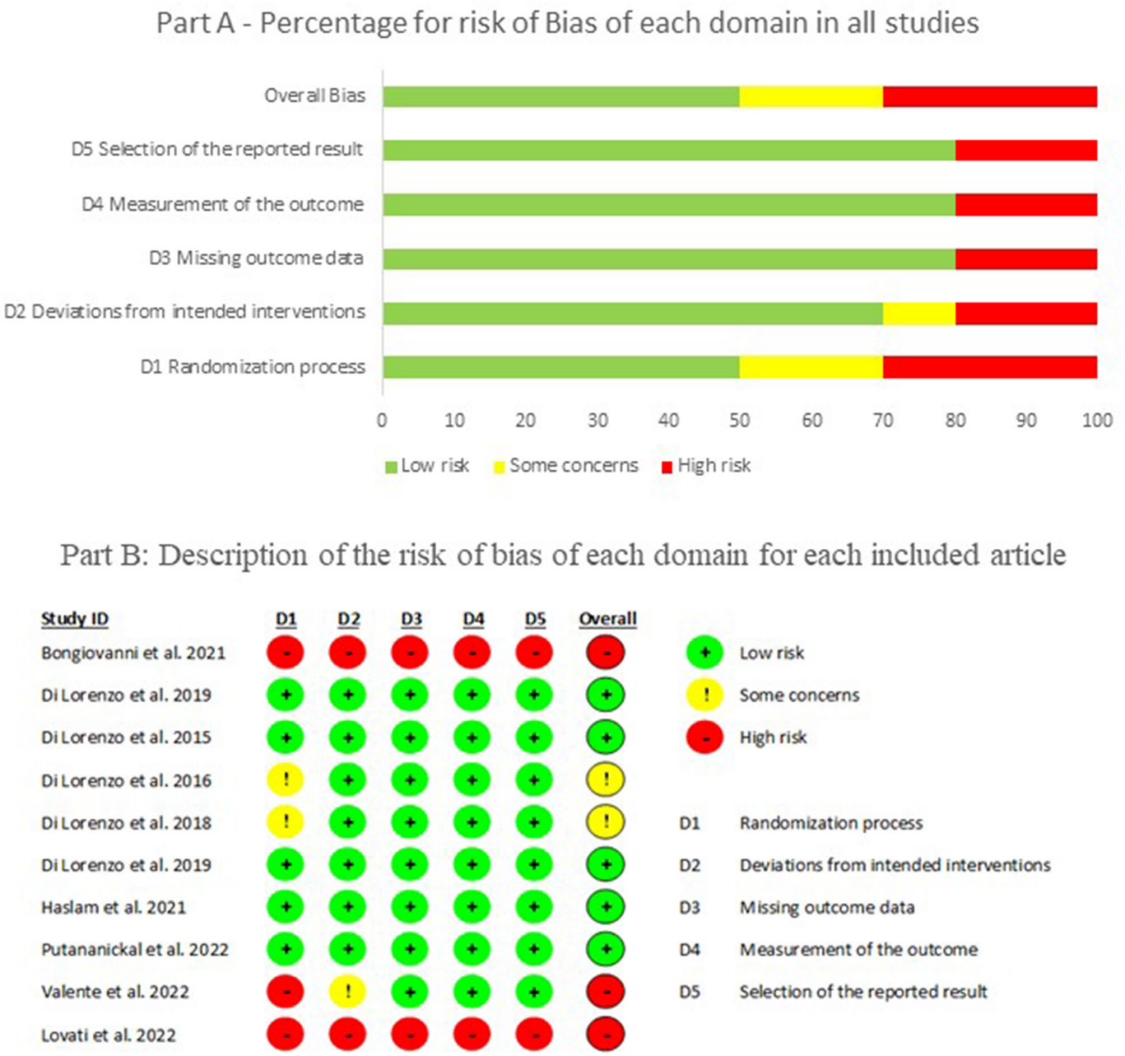
Risk of bias assessment according to the ROB 2.0 Cochrane tool, 2022.

The assessment of bias showed that 50% of the selected articles had a low risk of bias in all domains. The main domain with problems was the randomization process due to the methodological design of the studies, and few randomized controlled trials were included. Figure 3 shows the overall percentage of bias and for each domain according to the Rob 2.0 Cochrane tool (part A), along with detailed information on the risk of bias from each article (part B). This is reflected in the quality of the studies (MMAT), in which only two articles reached a high level of quality (five stars), and the majority received a three-star quality rating (60%), as can be seen in Table 2.

## 4. Discussion

This is the first attempt to systematically relate ketosis with migraine improvement through a meta-analysis of the effect of different ketogenic interventions (dietary or not) on migraine attacks. Even though the main result of the article is ketosis based, for the authors, it was challenging to standardize the pattern of results between articles, considering that most of them provide results with no mention of ketosis.

The results from this paper could support some beneficial effect of ketone bodies on the prevention or relief of migraine attacks, regardless of the strategy to increase ketosis. Unfortunately, the paucity and heterogeneity of available studies, which included only adult populations, does not allow the identification of demographic, clinical, or dietetic parameters that might correlate with a better outcome.

In stating the role of ketosis levels in migraine amelioration (mainly using BHB as a measure for KDT implementation), many red flags must be considered. First, some studies correlate the improvement of migraine attacks to weight loss in overweight subjects, probably related to a reduction in pro-inflammatory adipokines (39). Second, the correlation between blood and urine ketones is not well-defined and might vary depending on hydration/urine volume, acid-base balance, renal hemodynamics, and excretion (40). Third, KDT mechanisms of action might rely on both direct and indirect actions, such as modeling mitochondrial function, increasing glutathione, reducing ROS, and inhibiting the NLRP3 inflammasome (41).

Most of the articles included in this systematic review mainly addressed headache pain related to migraine (frequency, duration, and severity); however, some studies also measured aspects related to general disability (34, 37), medication use (29, 31, 37), or changes in BMI (29–31, 34).

Valente et al. (36) aimed to clarify whether the observed effect of KDT on migraine was due only to weight and fat mass reduction or not. Interestingly, patients benefitted from KDT independently from the reduced weight and fat mass, implying that the beneficial effects of KDT might be related to mechanisms other than just weight change (36). According to Lovati et al. (38), these possible mechanisms could be likely related to ketone production. In fact, Lovati et al. (38) found a correlation between urine ketone levels and migraine attack frequency (*p* = 0.0073), although in a limited sample (*n* = 13). By contrast, patients on a diet with a similar carbohydrate content but not ketogenic had no beneficial outcomes. Thus, these encouraging results should be viewed with caution because of the limited data available in the selected articles. In fact, the study by Haslam et al. (35), the only one that reported a ketosis level (only urinary), found no significant benefits from a KDT intervention nor found correlations between urinary ketosis and migraine severity.

Interestingly, there is no verified role for ketone levels in seizure control, even for epilepsy. Sharma et al. (42) recalled that, although some studies have demonstrated a positive effect between ketone levels in the blood and urine and seizure control, this finding has not been universal. In addition, it is possible that the age of the subject (42) and other variables may affect the correlation between seizure control and blood/urine ketone levels.

A dropout rate varying from 0 to 39% (average 21%) was reported in the included studies and was higher in patients undergoing cKDT (average 34%) and lower for the MAD (average 13%). As with epileptic patients, a lack of compliance can lie in many factors, with KDT inefficacy on disease symptoms as one of the most prominent. The meta-analysis of Ye et al. (43) on the efficacy and compliance to the cKDT and MAD in adult epileptic patients revealed an overall combined rate of compliance of 45%, with 38 and 56% compliance for cKDT and MAD, respectively. These findings are consistent with a previous study (44), in which compliance was significantly higher in patients undergoing the MAD than in patients following a cKDT. The dropout rate of 21% in the present study is not alarming when compared with the median of 30% of patients who report inconsistently adhering to recommended or prescribed acute migraine medication, or with the rate of only 24% of patients adhering to preventive medication (45). Future studies on migraine patients, both in the pediatric and adult age groups, should be designed to evaluate compliance rates according to migraine type and diverse ketogenic dietary interventions.

Many limitations of the present study must be considered. None of the selected studies included the pediatric population, making it impossible to assess KDT efficacy and tolerability in children/adolescents suffering from migraine. Only a few articles were considered eligible according to the inclusion criteria, the majority of which were from the same research group, potentially biasing the results. Moreover, the presence of bias was evidenced by the Rob2 instrument, especially due to the lack of randomization in most studies. Another limitation is the absence of regular ketosis measurement and the difficulty of assessing adherence to interventions.

Prospective randomized controlled trials are needed to confirm the efficacy and tolerability of KDT in migraine patients, both in the pediatric and adult age groups, and to study its optimal duration, repeatability, feasibility in normal weight subjects, and association with conventional migraine prophylaxis. Additionally, it is essential to underline that the phenotypic expressions of migraine vary greatly in the pediatric population compared with the adult population, thus inclusion criteria and population characteristics must be carefully considered when comparing outcomes.

## 5. Conclusion

The initial findings of the present review support some benefit of metabolic ketogenic therapy in migraine and encourage further studies, especially randomized clinical trials with appropriate and standardized methodology. Our Systematic Review strongly suggests the inclusion of adequate measurement of ketone levels during ketogenic therapy to check adherence to the treatment and improve knowledge on the relationship between ketone bodies and efficacy. Thus, we can assert with greater certainty that the strategy of increasing ketone bodies in the body can bring benefits in the relief and prevention of migraine.

## Data availability statement

The original contributions presented in the study are included in the article/Supplementary material, further inquiries can be directed to the corresponding author.

## Author contributions

LN, CF, and AT: conceptualization. LN, CF, LP, and AT: methodology. LN, GC, LP, MG, CF, EP, AC, and AT: investigation. LN, CF, MG, GC, LP, AC, and AT: data curation. LN, AT, GC, and LP: writing—original draft preparation. LN, CF, MG, GC, AT, EP, AC, and LP: writing—review and editing. AT: supervision. All authors have read and agreed to the present version of the manuscript.
