# Supplementary material for: Ketosis and migraine: a systematic review of the literature and meta-analysis
Source: Front Nutr. 2023 Jun 12;10:1204700. doi: 10.3389/fnut.2023.1204700 (PMC10292926; doi:10.3389/fnut.2023.1204700)
Supplement: Supplementary file 1 [file Table_1.DOCX]

Supplementary Material

Ketosis and migraine: systematic review of literature and meta-analysis

Lenycia de Cassya Lopes Neri, Cinzia Ferraris*, Guido Catalano, Monica Guglielmetti, Ludovica Pasca, Elena Pezzotti, Adriana Carpani, Anna Tagliabue

*** Correspondence:** Corresponding Author: cinzia.ferraris@unipv.it

# Supplementary Table: Search builders according database, limited for the last 10 years of publication.

| **Data base** | **Search strategy** |
| --- | --- |
| Pubmed | (((((((((("headache disorders"[MeSH Terms]) OR "Cephalgia"[All Fields]) OR "Cephalgias"[All Fields]) OR "Cephalalgia"[All Fields]) OR "Cephalalgias"[All Fields]) OR "headache"[MeSH Terms]) OR "headache"[All Fields]) OR "migraine disorders"[MeSH Terms]) OR "migraine"[All Fields]) OR "migraines"[All Fields]) AND ((((((((((((("ketones"[MeSH Terms]) OR "3-hydroxybutyric acid"[All Fields]) OR "ketone bodies"[All Fields]) OR "acetoacetates"[MeSH Terms]) OR "Ketosis"[MeSH Terms]) OR "Ketosis"[All Fields]) OR “Ketoacidos*”[All Fields]) OR “Metabolic Keto*”[All Fields]) OR “ Acetonemi*”[All Fields]) OR “ketonemi*”[All Fields]) OR “ketoacidemi*”[All Fields]) OR “ketonuri*”[All Fields]) OR “ketoaciduri*”[All Fields]) OR “acetonuri*”[All Fields] |
| Scopus | TITLE-ABS-KEY ( "headache disorders" OR "Cephalgia" OR "Cephalgias" OR "Cephalalgia" OR "Cephalalgias" OR "headache" OR "migraine" OR "migraines" ) AND TITLE-ABS-KEY ("ketones" OR "3-hydroxybutyric acid" OR "ketone bodies" OR "acetoacetates" OR "Ketosis" OR "Ketosis" OR “Ketoacidos*” OR “Metabolic Keto*” OR “ Acetonemi*” OR “ketonemi*” OR “ketoacidemi*” OR “ketonuri*” OR “ketoaciduri*” OR “acetonuri*”) |
| Web of Science | TS=(("headache disorders" OR "Cephalgia" OR "Cephalgias" OR "Cephalalgia" OR "Cephalalgias" OR "headache" OR "migraine" OR "migraines") AND ("ketones" OR "3-hydroxybutyric acid" OR "ketone bodies" OR "acetoacetates" OR "Ketosis" OR "Ketosis" OR “Ketoacidos*” OR “Metabolic Keto*” OR “ Acetonemi*” OR “ketonemi*” OR “ketoacidemi*” OR “ketonuri*” OR “ketoaciduri*” OR “acetonuri*”)) |
| Lilacs | tw:((tw:(("headache disorders" OR "Cephalgia" OR "Cephalgias" OR "Cephalalgia" OR "Cephalalgias" OR "headache" OR "migraine" OR "migraines" OR "cefalea" OR "cefaleas" OR "cefaleia" OR "cefaleias" OR "cefalalgia" OR "cefalalgias" OR "migraña" OR "migrañas" OR "migrañoso" OR "migrañosos" OR "dor de cabeça" OR "dores de cabeça" OR "dolor de cabeza" OR "dolores de cabeza" OR "Enxaqueca" OR "enxaquecas"))) AND (tw:(( "ketones" OR "3-hydroxybutyric acid" OR "ketone bodies" OR "acetoacetates" OR "Ketosis" OR "Ketosis" OR “Ketoacidos*” OR “Metabolic Keto*” OR “ Acetonemi*” OR “ketonemi*” OR “ketoacidemi*” OR “ketonuri*” OR “ketoaciduri*” OR “acetonuri*” OR "corpos cetônicos" OR "cetose" OR “cetosis" OR "Cuerpos Cetónicos” OR "acetoacetatos" OR "Cetosis" OR "Cetose" )))) |
| Science Direct | ("headache disorders" OR "Cephalgia" OR "Cephalalgia" OR "headache" OR "migraine") AND ("ketone bodies" OR "Ketosis" OR "3-hydroxybutyric acid " OR "acetoacetates") |

**
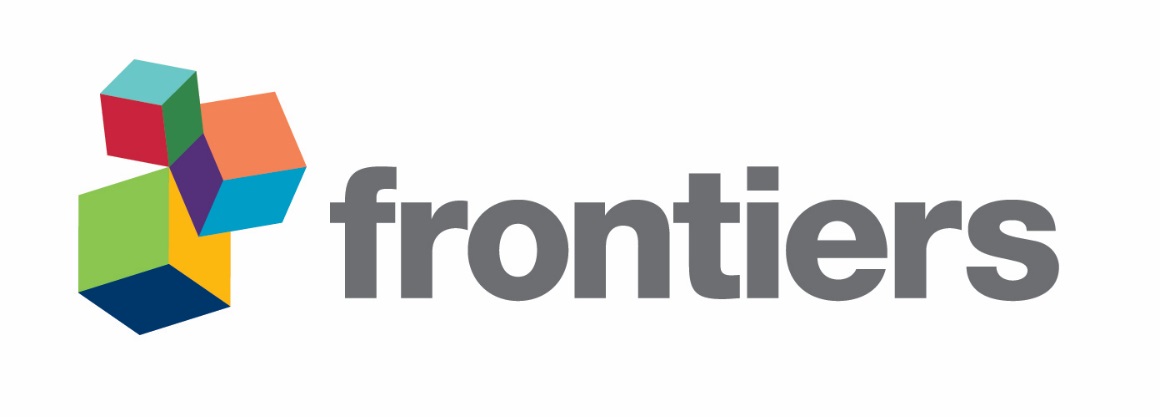
**
